# Evolutionary History of Assassin Bugs (Insecta: Hemiptera: Reduviidae): Insights from Divergence Dating and Ancestral State Reconstruction

**DOI:** 10.1371/journal.pone.0045523

**Published:** 2012-09-28

**Authors:** Wei Song Hwang, Christiane Weirauch

**Affiliations:** Department of Entomology, University of California Riverside, Riverside, California, United States of America; George Washington University, United States of America

## Abstract

Assassin bugs are one of the most successful clades of predatory animals based on their species numbers (∼6,800 spp.) and wide distribution in terrestrial ecosystems. Various novel prey capture strategies and remarkable prey specializations contribute to their appeal as a model to study evolutionary pathways involved in predation. Here, we reconstruct the most comprehensive reduviid phylogeny (178 taxa, 18 subfamilies) to date based on molecular data (5 markers). This phylogeny tests current hypotheses on reduviid relationships emphasizing the polyphyletic Reduviinae and the blood-feeding, disease-vectoring Triatominae, and allows us, for the first time in assassin bugs, to reconstruct ancestral states of prey associations and microhabitats. Using a fossil-calibrated molecular tree, we estimated divergence times for key events in the evolutionary history of Reduviidae. Our results indicate that the polyphyletic Reduviinae fall into 11–14 separate clades. Triatominae are paraphyletic with respect to the reduviine genus *Opisthacidius* in the maximum likelihood analyses; this result is in contrast to prior hypotheses that found Triatominae to be monophyletic or polyphyletic and may be due to the more comprehensive taxon and character sampling in this study. The evolution of blood-feeding may thus have occurred once or twice independently among predatory assassin bugs. All prey specialists evolved from generalist ancestors, with multiple evolutionary origins of termite and ant specializations. A bark-associated life style on tree trunks is ancestral for most of the lineages of Higher Reduviidae; living on foliage has evolved at least six times independently. Reduviidae originated in the Middle Jurassic (178 Ma), but significant lineage diversification only began in the Late Cretaceous (97 Ma). The integration of molecular phylogenetics with fossil and life history data as presented in this paper provides insights into the evolutionary history of reduviids and clears the way for in-depth evolutionary hypothesis testing in one of the most speciose clades of predators.

## Introduction

Assassin bugs (Hemiptera: Reduviidae) are the largest clade of predatory non-holometabolous insects (∼6,800 described species) [1], [2] and one of the largest clades of predatory animals. In addition, Reduviidae have adapted to a wide range of terrestrial habitats and diversified in their prey choices while developing a wide repertoire of innovative prey capture strategies [3], [4], [5], [6], [7]. Some Emesinae, the thread-legged bugs, cut through webs to reach their spider prey [3] or lure spiders using aggressive mimicry [4]. Apiomerini, Ectinoderini and Diaspidiini (Harpactorinae) coat their fore legs with plant resins for prey capture [5], while some members of the Harpactorini have evolved their own sticky secretions for the same purpose [6]. Holoptilinae, the feather-legged bugs, attract ants to imbibe paralyzing secretions before killing their prey [7]. The most infamous assassin bugs belong to the mostly Neotropical subfamily Triatominae, the kissing bugs, which feed on vertebrate blood. After humans colonized the Americas, several kissing bug species have adapted to blood-feed on humans where they vector *Trypanosoma cruzi* Chagas, the etiologic agent of Chagas disease [8]. Due to this range of predatory lifestyles and to the size of the group, assassin bugs offer a unique opportunity to investigate the evolution and diversification of one of the most speciose clades of animal predators. No study has so far addressed the evolutionary history of microhabitat and prey choices or examined the timing of key transitions within assassin bugs. We here present the largest molecular phylogeny of Reduviidae published to date with extensive subfamily representation and dense sampling of the polyphyletic Reduviinae. Based on this phylogeny, we trace the evolution of microhabitat colonizations and prey specialization within the group, but also date important diversification events based on a fossil-calibrated molecular divergence tree.

Assassin bugs are found in many terrestrial ecosystems and microhabitats, ranging from mammal burrows in the Sonoran desert to decomposing logs in the Bornean rainforest [9], [10]. Microhabitats of various assassin bug species are relatively well documented in the literature and are supplemented with our lab’s field observations. Interestingly, a large number of species are either found in association with the bark of trees or dwell on foliage of herbs, shrubs, and trees [11], [12], [13]. Several lineages of the Phymatine Complex (Centrocnemidinae, Elasmodeminae, Hammacerinae, Holoptilinae, Phymatinae) [14], [15], [16], the sister group to a clade that comprises the majority of Reduviidae, the “Higher Reduviidae”, are associated with the bark of trees and this association also occurs in various lineages within the Higher Reduviidae, which may infer that this association is ancestral for assassin bugs. Vegetation dwelling as a lifestyle, in contrast, occurs in more derived clades, e.g., the Phymatini among the Phymatinae and the Harpactorini among the Harpactorinae, and might therefore represent a derived microhabitat associations. We here test if bark association may represent the ancestral microhabitat for Reduviidae and trace microhabitat evolution across the group.

Ecological specializations have frequently been postulated to represent evolutionary dead-ends due to higher extinction risks [17], [18], [19], although this hypothesis has been challenged by some authors [20], [21]. According to this theory, specialist predation strategies would be more likely to evolve from generalist strategies, than the reverse transition from specialist to generalist predation. Assassin bugs show a pattern of generalist and specialist species, with some taxa apparently feeding on a wide range of prey species and others being specialized on certain taxonomic groups [12], [22]. Some of the most speciose clades within Reduviidae, such as the millipede-feeding Ectrichodiinae (>600 spp.), are specialists, while other specialist clades are much less diverse, e.g., the ant-feeding Holoptilinae (78 spp.) and the termite-specialist Salyavatinae (99 spp.) [1], [12]. Conversely, Harpactorinae (>2,000 spp.), the largest subfamily of Reduviidae, consists predominantly of generalist predators [11], [13]. We compiled feeding records of Reduviidae from the literature and our own observations to investigate evolutionary patterns across the phylogeny. Compared to the microhabitat dataset, the feeding dataset is less complete due to the scarcity of feeding observations in the laboratory and field. The assembled data together with the phylogeny nevertheless allow us to reconstruct generalist-specialist patterns, test whether reduviids evolved from an ancestral generalist or specialist predator, determine if reversals from specialization to generalist feeding have occurred, but also to predict feeding patterns for taxa with unknown feeding habits.

The phylogenetics of blood-feeding Triatominae has received considerable attention due to the epidemiological significance of certain species as vectors of Chagas disease in Latin America [23], [24], [25]. Conflicting hypotheses support Triatominae as monophyletic [14], [15], [24], [25] or propose polyphyletic origins for the blood-feeders [23], [26]. These alternative relationships impact interpretations of hematophagy in Reduviidae as a unique evolutionary event or as multiple independent evolutionary transitions. Schofield [23], [27] proposed multiple transitions to hematophagy and postulated a step-wise ecological scenario of separate lineages of predatory assassin bugs exploiting nest-dwelling invertebrates as a precursor to feeding on the vertebrate hosts. Almost all published triatomine phylogenies are based only on Triatomini and Rhodniini and exclude the remaining three triatomine tribes Alberproseniini, Bolboderini, and Cavernicolini [23], except Patterson and Gaunt [24], who reported a sister-group relationship between Bolboderini and Rhodniini. We here test relationships of Triatominae with the predatory Reduviidae by including 13 species of Cavernicolini, Triatomini and Rhodniini in the first multi-gene analysis that includes three triatomine tribes. We exclude Bolboderini and Alberproseniini due to the lack of data. Microhabitat and prey specialization of Triatominae and closely related reduviid species are traced to test if Schofield’s ecological scenarios are corroborated by our phylogenetic investigations.

Our current understanding of the evolutionary history of assassin bugs from fossils is based on a relatively small published fossil record that comprises 52 species (EDNA database http://edna.palass-hosting.org/, [28], [29]). Of these fossils, 31 are of questionable classification due to the lack of illustrations and meaningful descriptions. Reduviidae are relatively old, with one fossil that has been attributed to the Reduvioidea (Reduviidae + Pachynomidae) from the Early Jurassic and three reduviid specimens from the Early Cretaceous [30]. Fossils that can be reliably classified to subfamily, tribe, or genus are predominantly from Dominican and Baltic amber (Miocene – Eocene) and offer little insight into the evolutionary timing of major lineage diversification events within Reduviidae. In order to date some of these key events, we here use, for the first time in assassin bugs, divergence time estimates based on relaxed clock models and model calibration using described fossil taxa [31], [32], [33]. The use of fossil-calibrated molecular phylogenies in Hemiptera is in its infancy and currently restricted to agriculturally important Sternorrhyncha (psyllids [34], aphids [35]), some Auchenorrhyncha (cicadas [36], spittlebugs [37]) and one study on heteropteran infraorders [38]. Within Heteroptera, divergence times have so far only been investigated for Cimicoidea [39]. Previous molecular dating work within Reduviidae is restricted to a small data set, in terms of taxa and genes, of Triatominae and has used a strict-clock model [24], [40].

Recent phylogenetic analyses have recovered the monophyly of many, but not all, reduviid subfamilies while the monophyly of Reduviidae is well-established and Reduvioidea (Reduviidae + Pachynomidae) are sister-group to the rest of Cimicomorpha based on morphology [14], [15], [41], [42]. A notable exception are the Reduviinae, the second largest assassin bug subfamily, with worldwide 142 genera and ∼1,100 described species [1], which have long been suspected to be polyphyletic. Usinger [43], based on a ‘pre-cladistic’ phylogeny of Reduviidae, postulated that Reduviinae are ‘an unnatural group’ due to the fact that several genera were removed from that group to serve as type genera of new reduviid subfamilies, among them the Cetherinae, Vesciinae, and Sphaeridopinae. Due to the limited sampling of Reduviinae in previous analyses [14], [15], the extent of the reduviinae polyphyly problem remains in the dark. Our current analyses include an extensive sample of Reduviinae, allowing for tests of relationships and determining the major clades of Reduviinae. We regard our results as the first step towards resolving the Reduviinae polyphyly problem that will eventually lead to a re-classification of Reduviidae.

## Materials and Methods

### Taxon Sampling

A total of 178 taxa were sampled comprising 170 ingroup (Reduviidae) and 8 outgroup taxa (Nepomorpha: Belostomatidae, Corixidae; Pentatomomorpha: Scutelleridae, Aradidae; Cimicomorpha: Nabidae, Tingidae, Miridae). Ingroup sampling comprised 12 taxa of the Phymatine Complex (Centrocnemidinae, Elasmodeminae, Hammacerinae, Holoptilinae, Phymatinae); the remaining taxa belong to a clade that we here refer to as the ‘Higher Reduviidae’ (all Reduviidae with the exception of the Phymatine Complex). We recognize 25 subfamilies within the Reduviidae [1], [44], [45], 18 of which are represented in our analysis (Table S1). Taxa not included due to the lack of DNA quality material are the reduviid sister-group Pachynomidae and the assassin bug subfamilies Bactrodinae, Chryxinae, Elasmodeminae, Manangocorinae, Phimophorinae, Pseudocetherinae, and Sphaeridopinae. We included 75 terminal taxa (31 genera) of Reduviinae to test relationships of clades currently classified within this polyphyletic subfamily. Table S1 summarizes classification, molecular data, GenBank accession numbers, microhabitat, and prey specialization.

### Specimen Identification, Databasing, and Vouchering

Specimens were identified using species descriptions, identification keys e.g., [8], [46], [47] and images of type specimens where available. Undescribed species are listed as “n. sp.”, while specimens that could not be identified with certainty to species level are referred to as “sp.” or denoted as “nr. xxx” to the closest matching species. Inability to identify most species is due to the lack of adequate diagnoses and descriptions, illustrations and keys in historical literature. One hind leg was removed for non-destructive DNA extraction and subsequently mounted with the voucher specimen. Unique specimen identifier matrix bar-code labels (USIs) were associated with each voucher. Specimens were databased using the online specimen database of the Plant Bug Planetary Biodiversity Inventory (PBI) project (https://research.amnh.org/pbi/locality). Geo-referenced localities and other specimen information (e.g., images) are publicly available on the Discover Life website (http://www.discoverlife.org). Voucher specimens depository information is listed in Table S1.

### Molecular Markers and Primers

Five molecular markers were amplified comprising four ribosomal gene regions (16S rDNA, 18S rDNA, 28S D2 rDNA, 28S D3-D5 rDNA) and one nuclear protein-coding gene (wingless, Wg). The choice of the wingless gene is based on its utility for higher level phylogenetic studies of insects, especially Hemiptera [37], [48], [49], [50] and variation across Reduviidae is found to be at a suitable level (average 18.43%, range 15.36%–30.96%). For primer information and PCR thermocycling regimes see Weirauch and Munro [15] for ribosomal genes and Urban and Cryan [48] for the wingless gene.

### DNA Extraction, Amplification, Purification, and Sequencing

DNA was extracted using Qiagen DNeasy Blood and Tissue Kit standard protocols (Qiagen, Valencia, CA). Proteinase K digestion for dry specimens (see Table S1) was extended to 48 hrs. PCR amplification was conducted using Illustra PuReTaq Ready-To-Go PCR beads in an Eppendorf Thermocycler. Amplification results were visualized via gel electrophoresis with SyberSafe gel staining and UV illuminator. PCR products for ribosomal genes were purified using SureClean (Bioline); Wg PCR products encountered lower success rates in overall PCR amplification (see table S1) and required gel extraction using QIAquick Gel Extraction Kit standard protocols. Sanger (BigDye) DNA sequencing was conducted at the UCR Genomics Core facility. Sequences are deposited in GenBank (Table S1). Completeness of the molecular data set is 79.78%.

### Sequence Alignment and Phylogenetic Analysis

Sequences were edited and concatenated using Sequencher 4.8. Stop codons in open reading frames of Wg were checked in Sequencher. Sequences were aligned individually with MAFFT [51] (E-INS-i, G-INS-i, L-INS-i, Q-INS-i) and MUSCLE [52] to compare effects of alignment on phylogenetic analyses. SequenceMatrix 1.7.8 [53] and Mesquite 2.74 [54] were used to concatenate aligned gene regions into a combined molecular dataset. Lengths of the combined, aligned dataset ranged from 3,793 bp (E-INS-i) to 4,043 bp (Q-INS-i) (Table S2).

Phylogenetic analyses were conducted using TNT version 1.1 [55] (parsimony [P]) on a PC and RAxML-HPC2 [56] (maximum likelihood [ML]) on the teragrid accessible through the CIPRES web portal (http://www.phylo.org). TNT was set at 50, 80 and 100 initial levels to test the robustness of the search. All runs set at 80 and above produced identical results. Heuristic searches were conducted using New Technology Search with ratchet, tree-drifting, sectorial search, and tree-fusing with default settings. Best score hits of 10 times were performed and 500 standard bootstrap replicates were conducted. Internal gaps were treated as fifth character states in parsimony analyses, with terminal gaps converted to missing data. RAxML analyses used a partitioned dataset (i.e., treating the 5 gene regions separately) and rapid bootstrapping with automatic halt and subsequent higher bootstrap iterations (500–1,000). Support values are reported in the text henceforth in parentheses indicating the method of analysis (P for parsimony, ML for Maximum Likelihood). For bootstrap support, we define values >90% as strongly supported, 90–70% as well-supported/moderate support, <70% as weakly supported.

The different alignment strategies resulted in largely identical tree topologies in the RAxML analyses (Table S3). Bootstrap support values varied slightly between alignments (Table S3). Well-supported clades (>70%) were consistently recovered from all alignments. The MAFFT G-INS-i and MAFFT E-INS-i recovered identical topologies and only slight differences in bootstrap support values. The phylogenies discussed in the following are based on the MAFFT G-INS-i algorithm that shows highest congruence with published phylogenies [15]. For the MAFFT G-INS-i alignment we report 1,649 parsimony informative characters out of a total of 3,796 characters.

### Trait Evolution

Ancestral states for prey specializations and microhabitats, as separate characters, were reconstructed in Mesquite 2.74 using a parsimony model with characters treated as unordered and in BayesTraits 1.0 (www.evolution.rdg.ac.uk) for a maximum likelihood model [57]. We used the BayesMultistate method within BayesTraits with restrictions of equal probability for all state changes to reflect the one parameter Mk1 model for both microhabitat and prey specialization analyses. We based ancestral state reconstructions on the topology of the best likelihood tree from the RAxML analysis. Sources of data for prey specialization and microhabitat are listed in Table S1 and References S1. We coded terminal taxa based on biological data from congeneric species when observations for the species in the analysis were unavailable. We coded data as missing where genus-level data were unavailable.

### Molecular Dating

The divergence time estimate analysis was conducted using BEAST 1.6.1 [58] with a 4-gene partitioned dataset (16S rDNA, 18S rDNA, 28S rDNA, Wg), G-INS-i aligned, unlinked substitution models (GTR+Γ+I), relaxed clock uncorrelated lognormal, and 11 fossil data points for calibration. The 28S D2 and 28S D3–D5 gene regions were analyzed using the same clock model to reflect their single identity. The fossils were placed using the specimen-based method for placement within taxon groups (Table S4; [59]). We used the oldest-assigned fossil of the taxon which has unambiguous diagnostic characters to place it within a clade. Based on the geologic age range estimates provided by the fossil literature or updated estimates of the stratigraphy (Table S4), fossil ages were incorporated as taxon group priors with a lognormal distribution with a hard-bound minimum age and a soft-bound maximum age that captures the date range within the 95% confidence interval [60]. Ten million generations were performed, sampling every 1,000 generations to produce 10,000 trees. The initial 2,500 trees (25%) were discarded as burn-in using TreeAnnotator 1.6.1 [58]. The remaining 7,500 trees were used to produce the maximum clade credibility tree visualized using FigTree 1.3.1 (http://tree.bio.ed.ac.uk/software/figtree/).

## Results

### Phylogenetic Analyses

**Figure 1:**
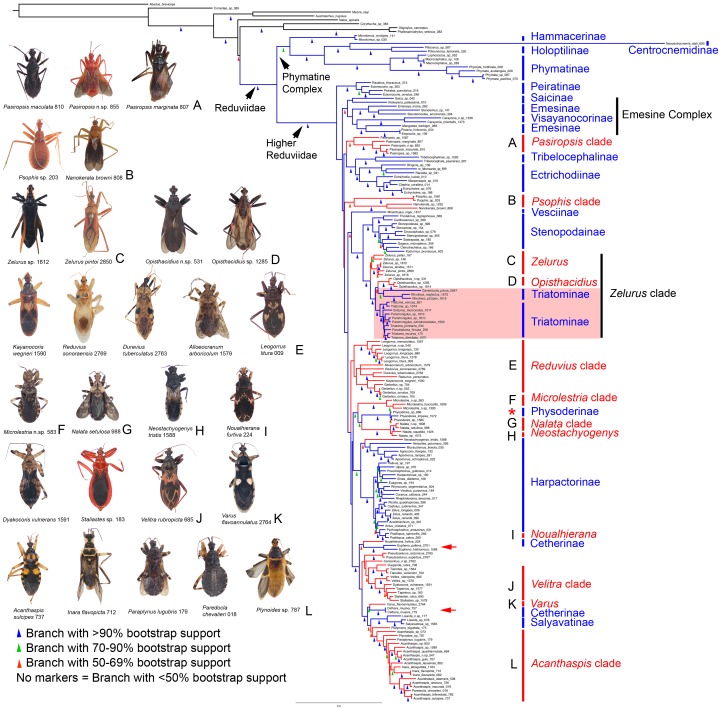
Maximum Likelihood phylogram with representative habitus images of reduviine clades. Best tree (score = −83447.290932) based on RAxML analysis of 178 taxa using a partitioned molecular dataset of 5 gene regions (16S, 18S, 28S D2, 28S D3–D5, Wg) aligned with MAFFT G-INS-i. Bootstrap values are indicated on branches by colored triangles according to support strength (explained by inset). Reduviinae lineages are indicated as red branches and remaining reduviids as blue while outgroup taxa are black. Habitus images of Reduviinae species with RCW specimen ID numbers are grouped (A–L) according to the 11 separate reduviine clades. The shaded red box highlights members of the hematophagous Triatominae, here shown as paraphyletic. Red arrowheads refer to the polyphyletic Cetherinae; the asterisk refers to Physoderinae nested within a reduviine clade.

**Figure 2:**
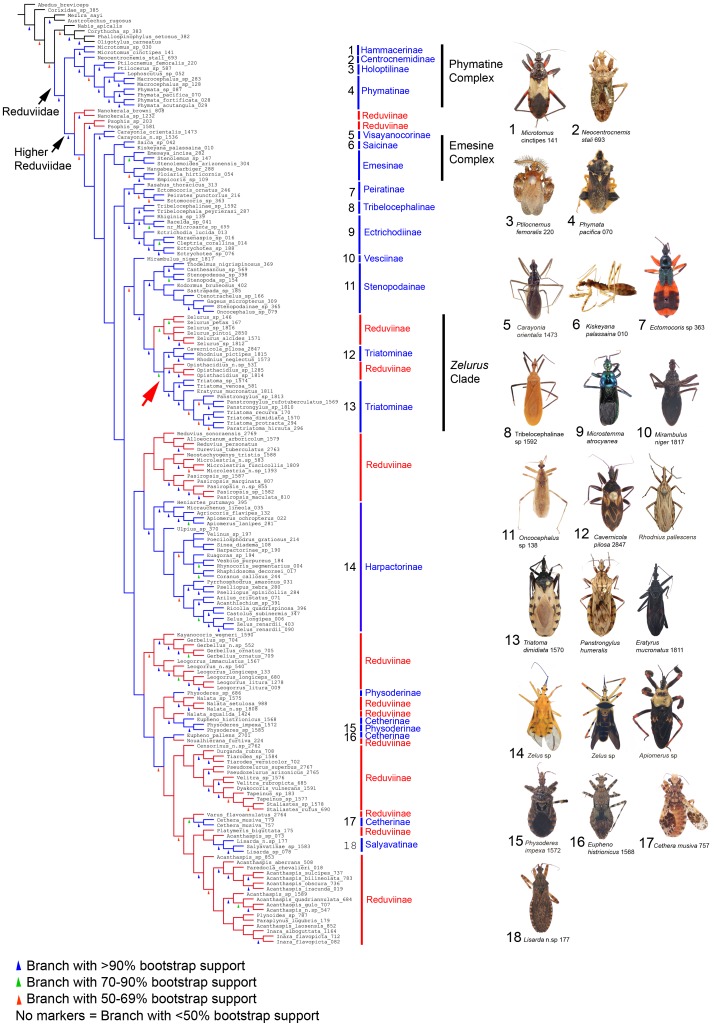
Strict consensus of 16 equally parsimonious trees with representative habitus images of reduviid subfamilies. Shortest trees (tree length = 23413, C.I. = 0.21, R.I. = 0.57) generated by TNT using the same molecular dataset (178 taxa, G-INS-i aligned, 5 gene regions) with bootstrap values indicated by colored triangles on branches (explained by inset). Reduviinae lineages are indicated as red branches and other subfamilies as blue while outgroup taxa are black. Habitus images of reduviids with RCW specimen ID numbers are labeled 1–18 according to subfamily membership indicated beside the phylogeny. Reduviinae are separated into 14 clades here and Triatominae + *Opisthacidius* form an unresolved polytomy (red arrowhead).

Figure 1 (ML; habitus images show the diversity in the subfamily Reduviinae) and figure 2 (P; habitus images show non-reduviine subfamilies) represent the largest, both in terms of terminals (178 taxa) and subfamily coverage (18 subfamilies), phylogeny of Reduviidae published to date. Although certain relationships above the subfamily level are weakly supported, these results drastically advance our understanding of assassin bug relationships and provide a solid framework for future studies. Most importantly, this analysis shows, for the first time, a glimpse of the true extent of the polyphyly of the large subfamily Reduviinae (lineages highlighted in red in Figs. 1 and 2). Hematophagous and disease vectoring Triatominae (red box in Fig. 1) are nested within a clade of large predatory Neotropical Reduviinae and are paraphyletic in the ML analysis due to the sister-group relationship of the reduviine *Opisthacidius* Berg and the triatomine Cavernicolini + Rhodniini. We further show that the rather unique big-eyed Cetherinae (red arrowheads in Fig. 1) are polyphyletic and split into an Old World and New World clade in the ML analyses. At a higher level, Reduviidae are monophyletic (P 96, ML 100) and the Phymatine Complex (P 95, ML 100) is consistently recovered as the sister to the Higher Reduviidae (P 93, ML 100), which include ∼90% of the reduviid species diversity. Sequence alignment data is provided in Table S2 with the resulting bootstrap support for clades of interest from topologies based on the different alignment methods summarized in Table S3.

#### Relationships within reduviidae

Within the Higher Reduviidae, the sister-group relationship between Ectrichodiinae and Tribelocephalinae is well-supported (P 94, ML 99). Similarly, a clade containing Stenopodainae, Triatominae, and the reduviine genera *Zelurus* Burmeister and *Opisthacidius* was consistently recovered with high support (P 99, ML 94). Most other relationships between subfamilies vary between analyses or receive weak support. We only highlight two of them: the “Emesine Complex” that we here define as comprising Emesinae, Visayanocorinae, and Saicinae, was recovered, with low support (ML 50), only in the ML analysis and is paraphyletic in the P analysis. Physoderinae (asterisk in Fig. 1) were grouped with *Microlestria* Stål and *Nalata* Stål in the ML analysis (ML 78), but are polyphyletic in the P analysis. In the Phymatine Complex, the long branch of *Neocentrocnemis stali* (Reuter) representing Centrocnemidinae is attributed to incomplete data (16S, Wg absent) due to suboptimal preservation of specimen. We retain this taxon in the phylogeny as its placement is consistent with previous analyses based on morphology and molecular datasets [14], [15]. No large insertions, deletions or highly divergent sequences are present in the ribosomal dataset of *N. stali* and therefore no long-branch attraction is suspected.

#### Monophyly of subfamilies

The monophyly of eight subfamilies was strongly supported in both P and ML analyses (Hammacerinae, Holoptilinae, Peiratinae, Phymatinae, Stenopodainae, Salyavatinae, Tribelocephalinae and Visayanocorinae). Two additional subfamilies were recovered as monophyletic with strong support in ML but not in P (Ectrichodiinae: ML 93, Physoderinae: ML 100). Saicinae were monophyletic only under ML, and merely with weak support (ML 44). Harpactorinae (P 62) and Emesinae (P<50) were monophyletic in the P analysis, but paraphyletic in the ML analyses. Cetherinae are polyphyletic, separating the Old and New World genera *Cethera* Amyot & Serville and *Eupheno* Gistel, respectively. Reduviinae are polyphyletic (see below) with all lineages nested within the Higher Reduviidae clade. Triatominae relationships are discussed below. The monophyly of Centrocnemidinae and Vesciinae was not tested due to single taxon representation.

#### Reduviinae polyphyly

Reduviinae are grouped into 11 (ML, Fig. 1) or 14 (P, Fig. 2) clades, some of which also include other subfamily-level taxa. Strongly supported clades (see Table S1 for membership of clades defined in this study) regardless of method used are the ‘*Velitra* clade’ (P 94, ML 100) and the ‘*Zelurus* clade’ (P 91, ML 96). We also recovered with strong support in ML but not in P, the ‘*Acanthaspis* clade’ (ML 97) and a more inclusive clade comprising Salyavatinae, the ‘*Acanthaspis* clade’, *Platymeris* Laporte, *Cethera*, and *Varus* Stål (P 59, ML 94). Some additional reduviine clades are recovered with weak support in ML, but are absent in the P analysis. These include the ‘*Psophis* clade’ (ML 59), the ‘*Reduvius* clade’ (ML 37), a clade comprising the *Velitra* clade and two additional reduviine genera, *Durganda* Amyot & Serville and *Tiarodes* Burmeister (ML 60), and the Old World Cetherinae *Cethera* grouping with *Varus* (P 59, ML 94). The monophyly of nine genera of Reduviinae was tested and recovered with strong to moderate support in both ML and P analyses (*Nanokerala* Wygodzinsky & Lent, *Psophis* Stål, *Microlestria*, *Gerbelius* Distant, *Leogorrus* Stål, *Opisthacidius*, *Pseudozelurus* Lent & Wygodzinsky, *Tiarodes*, *Velitra* Stål). *Pasiropsis* Reuter (P 93, ML 65) and *Zelurus* (P 70, ML 53) are weakly to strongly supported as monophyletic. *Nalata* (ML 100) and *Inara* Stål (ML 75) are strongly supported in ML but not in P. *Reduvius* Fabricius is paraphyletic with respect to *Durevius* Villiers. *Acanthaspis* Amyot & Serville is polyphyletic with several other reduviine genera nested within this genus (*Inara*, *Paraplynus* Schouteden, *Plynoides* Schouteden, *Paredocla* Jeannel); the monophyly of this more inclusive clade is strongly supported (see *Acanthaspis* clade above).

#### Triatominae relationships

Our analyses indicate a close relationship of Triatominae with the reduviine genera *Zelurus* and *Opisthacidius* (Figs. 1, 2). Rhodniini and Cavernicolini are strongly supported as sister taxa (P 98, ML 80) and Triatomini are monophyletic (P 98, ML 94). The subfamily Triatominae is paraphyletic with Triatomini being the sister-group to the *Opisthacidius* + (Rhodniini + Cavernicolini) clade in the ML analysis (Fig. 1). Parsimony analysis results in a polytomy of Triatomini, the Rhodniini + Cavernicolini clade and the *Opisthacidius* clade. *Triatoma* is polyphyletic in all our analyses, with *Paratriatoma* Barber, *Panstrongylus* Berg and *Eratyrus* Stål nested within this genus (Figs. 1, 2).

### Ancestral State Reconstructions of Microhabitats and Prey Specializations

**Figure 3:**
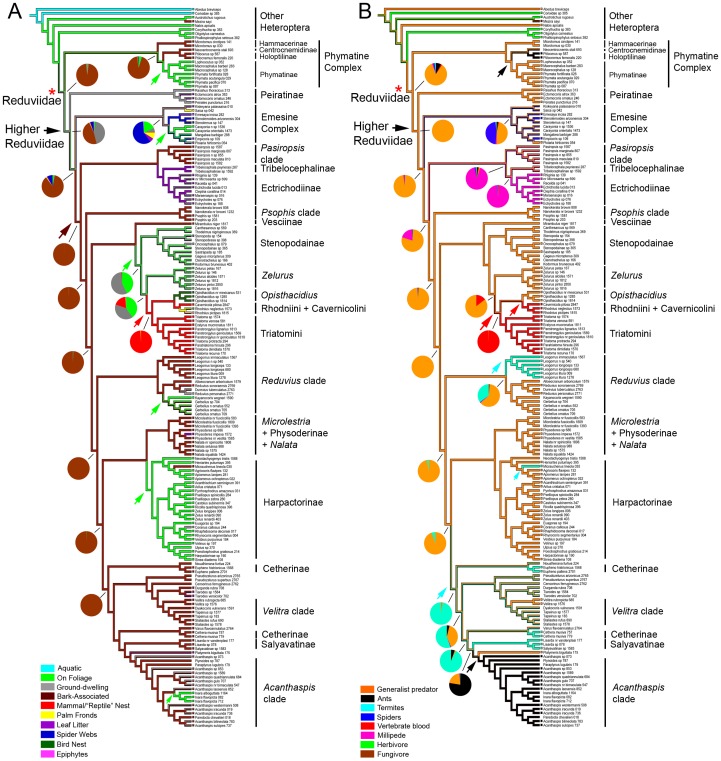
Ancestral state reconstructions based on best maximum likelihood tree. **A. Microhabitats.** Microhabitats of terminal taxa mapped onto ML best tree using Mesquite parsimony (P) model and maximum likelihood (ML) model in BayesTraits. Branches are color coded to represent different microhabitats (see color legends) based on parsimony and similarly-colored pie-charts represent probabilities generated from BayesTraits. Terminals without colored squares indicate unknown microhabitats and are coded as missing information in the matrix. Bark-associated lifestyle (brown arrowhead) is ancestral for all Higher Reduviidae except Peiratinae and Emesinae under both P and ML. Foliage-living (green arrowheads) has evolved at least six times independently within Reduviidae. Ancestral condition for all reduviids (red asterisk) remains ambiguous (bark associated/ground-dwelling/foliage-living) under P but ML favors bark-association (96.39%). Ancestral condition for Triatominae + *Opisthacidius* is mammal/“reptile” nest dwelling (red arrowhead). **B. Prey Specialization.** Prey specialization of terminal taxa mapped onto ML best tree using Mesquite parsimony (P) model and maximum likelihood (ML) model in BayesTraits. Branches and pie-charts (from ML) are color coded to represent different targeted prey (see color legends). Terminals without colored squares indicate unknown diets and are coded as missing information in the matrix. Ancestral condition for all reduviids is generalist predator (red asterisk). Hematophagy (red arrowheads) may have evolved once or twice independently under P while ML favors a single evolution (99.62%). Termite-specialization (cyan arrowheads) occurred at least three times independently while ant-specialization (black slanted arrowheads) evolved at least twice (Holoptilinae, *Acanthaspis* clade).

Our analysis shows multiple shifts between microhabitats at higher taxonomic levels, while closely related taxa, with a few exceptions, tend to share the same microhabitats (Fig. 3A). The evolutionary scenarios for the two most commonly encountered microhabitats – association with foliage versus tree bark – are quite different. Foliage was invaded at least six times independently by distantly related lineages (Fig. 3A; green arrowheads), including Emesinae, Harpactorinae, and Phymatinae. The bark-associated lifestyle in contrast is unambiguously optimized as the ancestral condition for most of the Higher Reduviidae (Higher Reduviidae except Peiratinae and the Emesine Complex; Fig. 3A; brown arrowhead) under both parsimony and maximum likelihood (99.21%–99.9% bark-associated) methods. Many clades within the Higher Reduviidae, especially among the Reduviinae lineages, retain this ancestral association. The maximum likelihood mapping projected bark-association as the most probable state (86.24%) for the ancestral nodes of the Higher Reduviidae including the Emesine Complex but excluding the Peiratinae while parsimony depicted this node as ambiguous between bark-association, living on foliage and ground-dwelling. A similar ambiguity is seen under parsimony for the ancestral state of Higher Reduviidae, while maximum likelihood predicted almost equal probabilities between bark-association (46.98%) and ground-dwelling (44.10%). The ancestral microhabitat for all Reduviidae (Fig. 3A; red asterisk) is ambiguous in the parsimony analysis, with possible microhabitats comprising the ground, tree bark or foliage of herbaceous vegetation or trees. The maximum likelihood method however placed bark-association as the most probable (96.39%) ancestral state for Reduviidae. The bark-associated lifestyle in some of the basal Reduviidae, the Hammacerinae, Centrocnemidinae, and some Holoptilinae, may thus either be homologous to the one in Higher Reduviidae, or may represent a separate colonization event from foliage or the ground, depending on the method used. Ground-dwelling habits (Fig. 3A; gray lineages) have evolved multiple times across Reduviidae and are frequently not inhabited exclusively, with taxa also recorded as inhabiting other microhabitats such as tree bark and foliage.

Mammal nests are here recovered as the ancestral microhabitats for blood-feeding Triatominae including the predatory reduviine species of *Opisthacidius* for both methods (96.52% ML). The three bat-feeding Triatominae, *Cavernicola pilosa* Barber, *Triatoma dimidiata* (Latreille) and *Eratyrus mucronatus* Stål, have colonized bat dwellings independently (data not shown, Fig. 3A). For the Emesine Complex, spider webs are reconstructed as the more likely ancestral habitat under maximum likelihood (59.42%) compared to foliage (17.68%), palm fronds (9.18%), ground (5.79%) and leaf litter (5.79%), whereas parsimony considered the node as ambiguous among these microhabitats (Fig. 3A).

Our reconstruction of prey preferences shows that the generalist predatory feeding strategy is ancestral for Reduviidae (84.93% ML; Fig. 3B; red asterisk) and that all prey specialists evolved from generalist ancestors (Fig. 3B; various cases across phylogeny). Ant specialization (Fig. 3B; black arrowheads) occurred twice independently among the included taxa Holoptilinae (75.97% ML), *Acanthaspis* clade (77.30% ML), while termite specialization evolved probably at least three times (Fig. 3B; cyan arrowheads) across Reduviidae (well documented in Salyavatinae and *Micrauchenus* Amyot & Serville, less well established in Cetherinae and *Leogorrus*). Millipede feeding is here shown to have evolved only once and can be traced to the base of the Ectrichodiinae unambiguously, or predicted to have occurred earlier at the Ectrichodiinae + Tribelocephalinae clade (97.13% ML) or even further to include the *Pasiropsis* sister-clade (91.55% ML). Prey preferences for the ectrichodiine sister-group Tribelocephalinae and *Pasiropsis* Reuter are unknown and it remains to be shown if the unique millipede association is shared with Tribelocephalinae and *Pasiropsis*. The reconstruction of spider specialization within Emesinae is ambiguous and either supports two independent origins or a single specialization event at the most recent common ancestor (46.51% ML). The transition from predatory to hematophagous life-style is ambiguous under parsimony, lending equal support to two scenarios on the evolution of blood feeding: 1) the switch to hematophagy may have occurred once at the base of the Triatominae + *Opisthacidius* clade (Fig. 3B; larger red arrowhead), with a reversion to generalist feeding behavior in *Opisthacidius*, or 2) Triatomini and Rhodniini + Cavernicolini may have acquired hematophagy independently (Fig. 3B; smaller red arrowhead). The maximum likelihood method overwhelmingly supports (99.62%) the first scenario of a single transition to hematophagy at the ancestral node of the Triatominae + *Opisthacidius* clade. The documentation of *Opisthacidius rubropictus* (Herrich-Schaeffer) in bird nests [61], presumably as an arthropod predator, also suggests two possible scenarios for the correlation between habitat switch and the transition from predatory to hematophagous habits: the colonization of vertebrate nests either preceded the evolution of hematophagy or it coincided with the transition to blood-feeding.

### Molecular Dating

**Figure 4:**
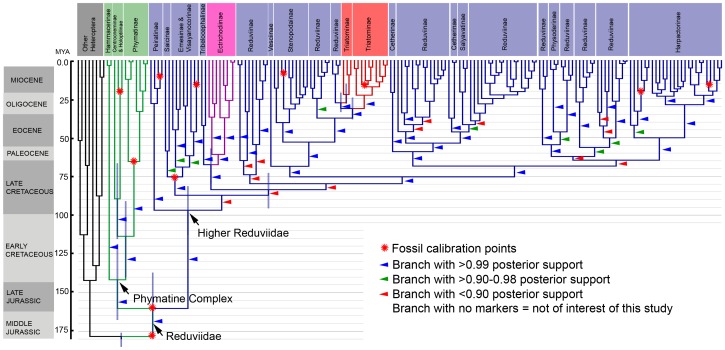
Divergence time estimates based on BEAST analysis using relaxed-clock model and 11 fossil calibration points. Chronogram based on same G-INS-i aligned molecular dataset (178 taxa; 5 gene regions: 16S, 18S, 28S D2, 28S D3–D5, Wg), using unlinked substitution models (GTR+Γ+I), relaxed clock uncorrelated lognormal and 11 fossils as priors. Lineages are colored on the chronogram as follows: Outgroup taxa (black), Phymatine Complex (green), Ectrichodiinae (pink), Triatominae (red), all other reduviid subfamilies (blue). Posterior probabilities are indicated on branches by colored triangles (see inset). Shaded node bars indicate 95% highest posterior density (HPD) credibility intervals for clades of interest only. Placement of fossils as calibration points of clades indicated by red stars.

The BEAST analysis produced a phylogeny that is highly congruent with the ML analysis (Fig. 4), but somewhat less similar to the topology of the P analysis. The monophyly of all strongly-supported major clades and subfamilies is recovered as well as the paraphyly of Triatominae and the polyphyly of Cetherinae. The origin of Reduviidae is dated to 178 Ma [176–185 Ma] and thus falls within the Middle Jurassic (Fig. 4, Table S5). The divergence between the Phymatine Complex and the Higher Reduviidae occurred shortly thereafter, at around 160 Ma (137–180 Ma) during the Late Jurassic. The diversification of the Higher Reduviidae began only in the Late Cretaceous starting at 97 Ma [81–113 Ma] and continued through the Miocene. The origins of all subfamily-level clades within the Phymatine Complex (Hammacerinae, Phymatinae, Centrocnemidinae, Holoptilinae) are comparatively older than all subfamily-level clades in the Higher Reduviidae with the exception of the Peiratinae. The oldest Phymatine Complex subfamily is Hammacerinae at 142 Ma (119–168 Ma) and the youngest is Holoptilinae and Centrocnemidinae at 90 Ma (67–115 Ma). Comparatively, the oldest Higher Reduviidae subfamily is Peiratinae at 97 Ma (81–113 Ma) and the youngest is Triatominae at 32 Ma (24–38 Ma). Chronogram with terminal taxon names and all 95% highest posterior density (HPD) node bars annotated is provided as Figure S1 and age estimates of selected clades are summarized in Table S4.

## Discussion

### Triatominae and the Origin of Blood-feeding in Reduviidae

With the extensive taxon sampling of Triatominae and related predatory Reduviidae, and the large and relatively complete set of sequence data, we here present the most rigorous test of triatomine monophyly or polyphyly published to date. As opposed to previous analyses [14], [15], [24], we did not recover a monophyletic Triatominae in any of our analyses, nor did we find support for Triatominae being polyphyletic [23], [27]. Instead, Triatominae are paraphyletic with respect to the genus *Opisthacidius* based on the ML analysis (Fig. 1) or part of a polytomy that also includes *Opisthacidius* in the P analysis (Fig. 2). Short branch lengths between *Opisthacidius*, the Rhodniini + Cavernicolini clade, and the Triatomini indicate that additional data is required to further test relationships among these three well-supported clades. Our results show that Triatominae are nested within the *Zelurus* clade that is restricted to the New World, supporting the hypothesis of a Neotropical origin of Triatominae [8], [25]. The existence of Old World triatomines, namely the South Asian *Linshcosteus* Distant and the South-east Asian *rubrofasciata* species complex of *Triatoma* Laporte has intrigued workers for the past two decades [8], [25], [62]. This disjunct distribution was even interpreted as support for the hypothesis of a polyphyletic Triatominae [62]. Although not included in our analyses, *Linshcosteus* and *T. rubrofasciata* have been placed within Triatomini [25] and our dating estimate for Triatomini (∼32 Ma) suggests that the Old World Triatomini represent a relatively recent dispersal rather than an older vicariant event.

Our divergence time estimates (Fig. 4) for Triatomini (32 Ma) and for Rhodniini + Cavernicolini (27.5 Ma) are much younger than the 107 Ma age that Patterson and Gaunt [24] postulated for Triatominae using a fixed molecular clock model. A strict clock analysis is shown to be accurate only for shallow phylogenies (Miocene and later) but not for cases where rate variation is higher [63]. Our use of a relaxed clock model for dating cladogenetic events among Triatominae is therefore a significant improvement, given that constant rate variation is implausible for deep divergences [64]. Based on our estimates, Triatominae evolved in the Oligocene when South America was already isolated from Antarctica and migrating towards North America [65], [66]. Our analysis therefore does not show a link between the evolution of triatomine hematophagy and the break-up of Gondwanaland as hypothesized by Patterson and Gaunt. Instead, we propose that the emergence of hematophagous triatomines in the Oligocene coincided with two other large-scale events: a period of well-documented species radiations among Neotropical mammals and birds [67], [68], [69] and a period of extensive diversification of ecotypes in South America then and thereafter [70], [71], [72].

The lack of well-defined host specificity between genera and species groups of Triatominae with their respective vertebrate hosts has long puzzled scientists [8], [73]. Vertebrate host associations are generally much more specific in other blood-feeding insects such as Phthiraptera (lice) [74], and Cimicidae (bedbugs) [75], which suggests a co-evolutionary history between the host and parasite. This is not the case for many Triatominae such as certain species of *Panstrongylus* and *Triatoma* that appear to feed indiscriminately on opossums, bats and other mammals [8]. Besides ecological factors that may determine host specificity, the relatively younger age of Triatominae (27–32 Ma) compared to lice 115–130 Ma [76] and bedbugs 100 Ma [39] may contribute to this lesser degree of host-parasite specificity observed in kissing bugs. Claims of any correlation between host and habitat diversification and co-speciation within Triatominae will also require denser taxon sampling and host-parasite co-evolutionary analyses.

The colonization of vertebrate nests occurred only once according to our analysis (Fig. 3A) and may be interpreted as a precursor for the evolution of hematophagy, although our optimization also allows for the possibility that nest invasion and switch to blood-feeding co-occurred (Fig. 3A, B; red arrowheads). The single nest colonization event may indicate that the transition from a free-living predatory to nest-inhabiting hematophagous lifestyle is less easily achieved in evolutionary terms than indicated by Schofield [27], who proposed multiple of such switches to have given rise to a polyphyletic Triatominae.

### Early Diversification Patterns of Reduviidae

Our divergence time estimates (Fig. 4) provide the first glimpses into the timing of evolutionary events in the second largest family of True Bugs, the Reduviidae, and is one of less than a handful of dating analyses for Heteroptera [24], [38], [39]. These estimates allow us to formulate explicit hypotheses on the timing of specific cladogenetic events that can be further investigated. One of these hypotheses is the early and continuous divergence of subfamily-level clades within the Phymatine Complex (Early Cretaceous) as opposed to the apparently delayed diversification (Late Cretaceous) within the Higher Reduviidae, a clade comprising ∼90% of the extant species diversity. The Late Cretaceous start of the Higher Reduviidae diversification coincides with two global changes affecting all terrestrial ecosystems, the radiations of angiosperms and phytophagous insects [33], [77], [78], [79], [80]. Both of these events have likely impacted the evolution of Reduviidae, by supplying increased microhabitat heterogeneity as well as new food sources for these predatory insects. The initial diversification of Higher Reduviidae occurred over a relatively short period of about 31 million years (65–96 Ma), which partially accounts for the lack of strong support for subfamily and higher-level clade relationships within Higher Reduviidae.

### Microhabitat Colonizations

The ability of reduviids to colonize a wide range of microhabitats (Fig. 3A) might be one of the factors that have influenced their high species diversity, driven by ecological adaptations. Of the six independent transitions to foliage-dwelling, three clades are noteworthy for their high species numbers: Phymatinae (291 spp. [2]), *Zelurus* (132 spp. [1]), and Harpactorini (∼2,000 spp. [1]). High species diversity is however not linked to this particular microhabitat, since Ectrichodiinae (>600 spp.; ground-dwelling/leaf-litter) and Stenopodainae (∼732 spp.; ground-dwelling/leaf-litter/on foliage) are both found in various microhabitats and are among the most speciose reduviid subfamilies (Fig. 3A). Conversely, some of the clades that have retained the ancestral bark-associated lifestyle among Higher Reduviinae are also speciose, best exemplified by the large *Acanthaspis* and *Velitra* clades. We suspect that factors other than microhabitat association may have driven the diversification of Reduviidae, among them prey specialization and changes in prey capture techniques.

Even though our analyses tend towards bark-association as the ancestral microhabitat of all Reduviidae (Fig. 3A, ML 96.39% bark-association, P ambiguous between bark-association, foliage-dwelling, ground-dwelling), this is not conclusive at the moment. The inclusion of members of the rarely collected ground-dwelling (pers. obs.) reduviid sister-group Pachynomidae [81], [82], [83] that were unavailable for this study will further test, and refine, this hypothesis.

### Prey Preferences

The hypothesis that specialized taxa are more susceptible to mass extinction events [19] and therefore more likely to be restricted to the tips of a phylogeny [18] is not entirely corroborated by our analysis of Reduviidae (Fig. 3B). Some specialized clades are relatively old (ant specialist Holoptilinae [90 Ma], millipede specialist Ectrichodiinae [67.5 Ma], spider specialist Emesinae [75 Ma]), but others are clearly more recently evolved specializations (blood-feeding Triatominae [32 Ma and 27.5 Ma], termite specialist Salyavatinae [42 Ma] and *Micrauchenus* [20 Ma], and the ant specialist *Acanthaspis* clade [25 Ma]) (Fig. 4). This indicates that specialized predators may not necessarily suffer a higher extinction risk due to a more restricted diet. Likewise, the hypothesis that prey specialization constrained food availability and therefore impacts the ability of specialists to diversify (e.g., [17]) is not corroborated by Reduviidae (Fig. 3B, [1]). We do however observe a general trend of specialists to evolve from generalist ancestors rather than the reverse as documented for some insects [20], [21].

Even though reduviids are currently mostly regarded as generalist predators, this observation might mostly be due to the limited number and nature of published observations documenting specialization (Table S1). We therefore expect that additional cases of prey specializations will be discovered as more detailed field observations and experiments become available.

### Reduviinae Polyphyly

Our extensive sampling of Reduviinae generates a phylogeny-informed framework for the eventual re-classification of this polyphyletic assemblage, a somewhat daunting task given the size of the group and the number of included genera. In 1904, Distant proposed a first classification of the group, referred to by him as Acanthaspidinae, and grouped 23 genera into 6 divisions [84] (Table S6), unfortunately without identifying diagnostic characters for these divisions. Unsurprisingly, Distant’s classification was not adopted by later workers and subsequently described reduviine taxa were not grouped accordingly. We here recognize the reduviine clades derived from our ML phylogenetic analysis (Fig. 1, clade membership listed in Table S1) and tentatively propose the inclusion of 45 additional genera that were not included in the current analysis based on similar general morphology (Table S7), with the remaining 64 unexamined reduviine genera listed as uncertain placement. The membership of these additional 45 taxa remains to be tested by future cladistic analyses that also should include morphological data to eventually generate meaningful diagnoses.

Interestingly, three among the proposed reduviine clades (*Acanthaspis* clade, *Reduvius* clade, *Velitra* clade) together represents 48% (525 spp.) of the entire reduviine diversity [1]. *Reduvius* and *Acanthaspis* are the most (197 spp.) and second most (110 spp.) speciose genera of Reduviinae, respectively [1], but neither one of them is monophyletic. Non-monophyly at the genus-level will complicate a future re-classification, since multiple species will have to be phylogenetically evaluated before a placement for the genus in question (or parts thereof) can be proposed. On a positive note, we believe that the Reduviinae polyphyly problem is now rather well defined, allowing for independent phylogenetic and taxonomic revisions of several smaller, more manageable clades.

### Conclusion

Employing molecular, fossil, microhabitat and prey specialization data, we present the first comprehensive hypothesis on the evolutionary history of Reduviidae. The inclusion of multiple Reduviinae taxa has significantly improved our notion of the overall Reduviidae phylogeny. Fossil-calibrated divergence time estimates indicate that the diversification pattern is different between the Phymatine Complex and the Higher Reduviidae, while more focal research on the early diversification of Higher Reduviidae is required to determine the deeper node relationships. We show that bark-associated living is an ancestral condition for most of Higher Reduviidae including all Reduviinae while living on foliage has evolved independently at least six times across Reduviidae. Prey specializations occur in old as well as more recent clades and have coincided with significant diversification in some cases such as the millipede-feeding Ectrichodiinae. More field observations across the family will enhance our understanding of both microhabitat and prey selection and provide a more accurate picture of their evolutionary pattern. Finally, we show a close relationship between the Neotropical reduviine genus *Opisthacidius* and the presumably paraphyletic hematophagous Triatominae and propose that the clade including these taxa has diverged relatively recently (∼32 Ma).

## Supporting Information

Figure S1 **Chronogram with terminal taxon names and 95% HPD node bars.**
(TIF)

Table S1 **Taxon list with ID numbers, depository information,** GenBank accession numbers, Reduviinae clade classification, microhabitat and prey specialization coding with references and locality information. Footnote: * refers to dried museum specimens.
(XLS)

Table S2 **Summary of individual gene region and combined sequence lengths of dataset based on different alignment algorithms.**
(XLS)

Table S3 **Table for bootstrap values of all subfamilies and Reduviinae clades based on different sequence alignment algorithms.**
(XLS)

Table S4 **Fossil calibration table with fossil taxonomic information, locality, taphonomy, fossil age and age references.**
(XLS)

Table S5 **Summary table of age estimates of selected reduviid clades with 95% highest probability density intervals.**
(XLS)

Table S6 **Distant’s 1904 classification of Reduviinae (Acanthaspidinae) into six divisions.**
(XLS)

Table S7 **Proposed clade-membership of Reduviinae genera.** Footnote: genera in bold font represent genera included in present study, genera in regular font are genera absent here.
(XLS)

References S1 **List of references (85–128) from which microhabitat and prey specialization information were derived and compiled as shown in Table S1.**
(DOC)
