# Supplementary figures and images for: Evolutionary History of Assassin Bugs (Insecta: Hemiptera: Reduviidae): Insights from Divergence Dating and Ancestral State Reconstruction
Source: PLoS One. 2012 Sep 28;7(9):e45523. doi: 10.1371/journal.pone.0045523 (PMC3460966; doi:10.1371/journal.pone.0045523)

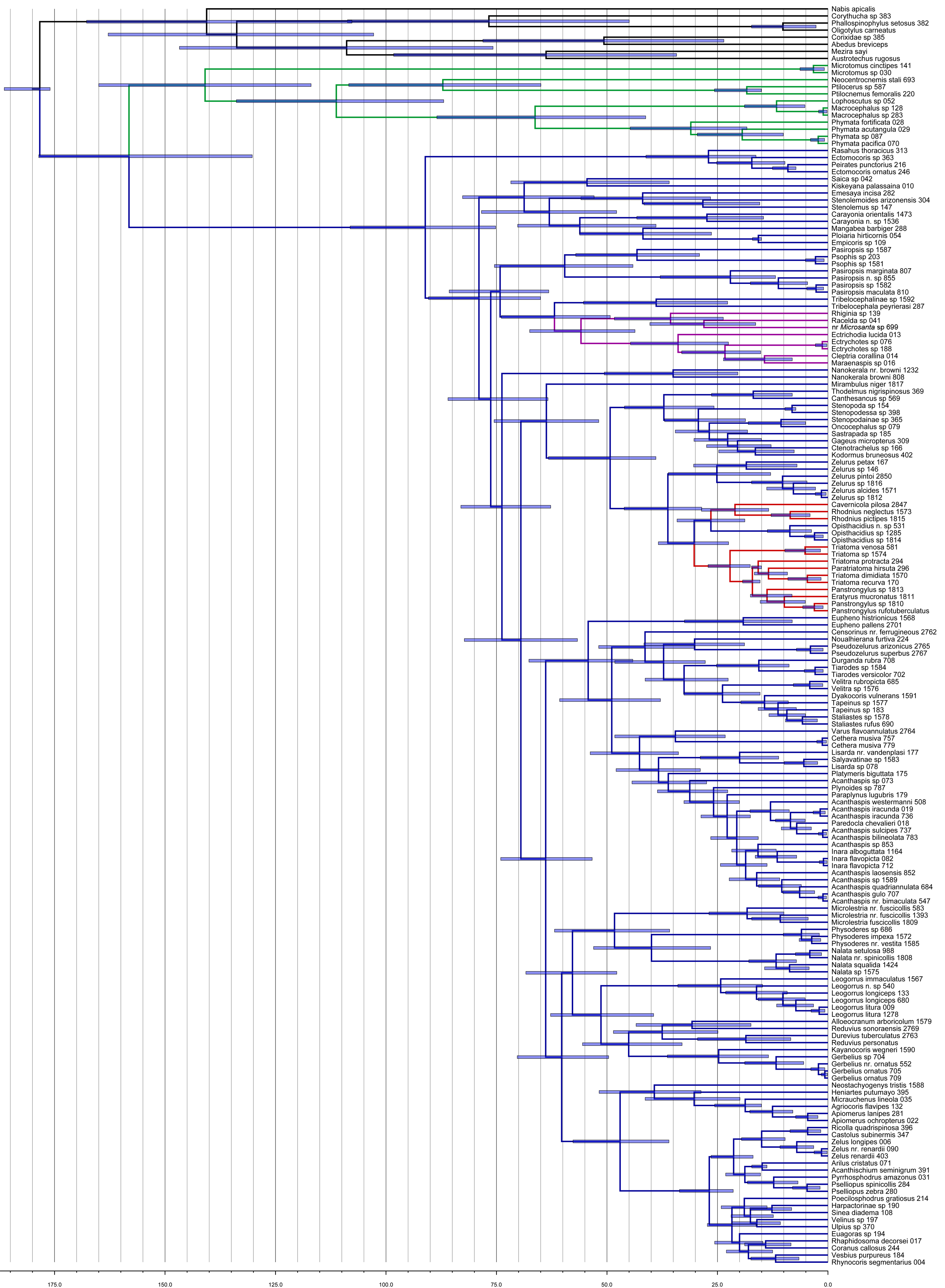

Supplement: Figure S1 — Chronogram with terminal taxon names and 95% HPD node bars. (TIF) [file pone.0045523.s001.tif]
