# Supplementary material for: Evolutionary History of Assassin Bugs (Insecta: Hemiptera: Reduviidae): Insights from Divergence Dating and Ancestral State Reconstruction
Source: PLoS One. 2012 Sep 28;7(9):e45523. doi: 10.1371/journal.pone.0045523 (PMC3460966; doi:10.1371/journal.pone.0045523)
Supplement: References S1 — List of references (85–128) from which microhabitat and prey specialization information were derived and compiled as shown in Table S1. (DOC) [file pone.0045523.s009.doc]

**References S1**

85. Cassis G, Vanags L (2006) Jewel bugs of Australia (Insecta, Heteroptera, Scutelleridae). Denisia 19: 275-398.

86. Usinger RL, Matsuda R (1959) Classification of the Aradidae (Hemiptera-Heteroptera). London: British Museum (Natural History). 410 p.

87. Taylor SJ, Gil SA (2009) State Records, Confirmations, and Habitats of Aradidae (Hemiptera: Heteroptera) from Louisiana, U.S.A. The Florida Entomologist 92: 199-207.

88. Nardi JB, Bee CM, Miller LA, Taylor SJ (2009) Distinctive features of the alimentary canal of a fungus-feeding hemipteran, *Mezira* *granulata* (Heteroptera: Aradidae). Arthropod Structure & Development 38: 206-215.

89. Lattin JD (1989) Bionomics of the Nabidae. Annual Review of Entomology 34: 383-400.

90. Wellhouse WH (1919) Lace bug on hawthorn, *Corythuca* *bellula* Gibson. Journal of Economic Entomology 12: (441-446).

91. Gibson EH (1918) The genus *Corythuca* Stal. Transactions of the American Entomological Society 44: (69-104).

92. Schuh RT (2000) Revision of *Oligotylus* Van Duzee with descriptions of ten new species from western North America and comments on *Lepidargyrus* in the Nearctic (Heteroptera: Miridae: Phylinae: Phylini). American Museum Novitates 3300: 1-44.

93. Weirauch C (2006) New genera and species oak-associated Phylini (Heteroptera : Miridae : Phylinae) from western North America. American Museum Novitates: 1-54.

94. Miller NCE (1956) Centrocneminae, a new sub-family of the Reduviidae (Hemiptera-Heteroptera). Bull Brit Mus [Nat Hist] Ent 4: 217-283.

95. Haviland MD (1931) The Reduviidae of Kartabo, Bartica District, British Guiana. Zoologica [New York] 7: 129-154.

96. Lawrence RF (1984) The centipedes and millipedes of southern Africa. A guide: A.A. Balkema, Rotterdam & Cape Town. 1-148 p.

97. Haridass ET (1985) Feeding and ovipositional behavior in some reduviids (Insecta, Heteroptera) Proceedings of the Indian Academy of Sciences-Animal Sciences 94: 239-247.

98. Wygodzinsky PW (1966) A monograph of the Emesinae (Reduviidae, Hemiptera). Amer Mus Natur Hist Bull 133: 1-614.

99. Malipatil MB (1985) Revision of Australian Holoptilinae (Reduviidae, Heteroptera). Australian Journal of Zoology 33: 283-299.

100. McKeown KC (1944) Australian Insects. An Introductory Handbook. Sydney: Royal Zoological Society of New South Wales. 304 p.

101. Champion GC (1899) Insecta Rhynchota. Hemiptera-Heteroptera, Vol II. Biologia Centrali Americana. London: Taylor & Francis. pp. 162-296.

102. Gil-Santana HR (2002) Predation of *Lagria* *villosa* Fabricius, 1783 (Coleoptera: Lagriidae) by *Apiomerus* *nigrilobus* Stal, 1872 (Hemiptera: Reduviidae: Apiomerinae) at Cabo Frio in the state of Rio de Janeiro, Brasil. Entomologia y Vectores 9: 201-208.

103. Koponen M (1988) Brief reports. Heteroptera. *Castolus* *tricolor* Champion (Reduviidae) found in Finland. Notulae Entomologicae 68: 150.

104. Wachmann E, Melber A, Deckert J (2006) Hemiptera Volume 1: Dipsocoromorpha, Nepomorpha, Gerromorpha, Leptopodomorpha, Cimicomorpha (part 1). Tierwelt Deutschlands 77: 1-263.

105. Sahayaraj K, Ambrose DP (1993) Biology and predatory potential of *Coranus* *nodulosus* Ambrose and Sahayaraj on *Dysdercus* *cingulatus* Fabr. and *Oxycarenus* *hyalinipennis* Costa (Heteroptera: Reduviidae). Hexapoda (Insecta Indica) 5: 17-23.

106. Wygodzinsky P (1948) Contribuicao ao conhecimento do genero *Heniartes* Spinola 1837 (Apiomerinae, Reduviidae, Hemiptera). Arq Mus Nac [Rio De Janeiro] 41: 9-64.

107. Berenger J-M, Pluot-Sigwalt D (2009) Notes on *Micrauchenus* *lineola* (Fabricius 1787), a termitophilous and termitophagous species (Reduviidae: Harpactorinae, Apiomerini). Annales De La Societe Entomologique De France 45: 129-133.

108. Gil-Santana HR, Valka Alves RJ (2011) Association between *Zelus* *versicolor* (Herrich-Schaeffer) (Hemiptera, Reduviidae, Harpactorinae) and *Bidens* *rubifolia* Kunth (Asterales, Asteraceae). EntomoBrasilis 4: 30-32.

109. Miller NCE (1956) The biology of the Heteroptera. London: L. Hill (Books) Ltd. 162 p.

110. Ambrose DP (1987) Biological, Behavioral, and Morphological Tools in the Biosystematics of Reduviidae (Insecta, Heteroptera, Reduviidae). Proceedings of the Indian Academy of Sciences-Animal Sciences 96: 499-508.

111. Kormilev NA (1981) On some Neotropical species of the genus *Macrocephalus* (Hemiptera: Phymatidae). Sociobiology 6: 214-220.

112. Balduf WV (1941) Life history of *Phymata* *pennsylvanica* *americana* Melin (Phymatidae, Hemiptera). Ann Ent Soc America 34: 204-214.

113. Balduf WV (1943) Third annotated list of *Phymata* prey records (Phymatidae, Hemiptera). Ohio Jour Sci 43: 74-78.

114. Balduf WV (1948) A summary of studies on the ambush bug *Phymata* *pennsylvanica* *americana* Melin (Phymatidae Hemiptera). Transactions of the Illinois Academy of Science 41: pp. 101-106.

115. Miller NCE (1954) New genera and species of Reduviidae from Indonesia and the description of a new subfamily (Hemiptera, Heteroptera). Tijdschrift voor Entomologie Amsterdam 97: 75-114.

116. Odhiambo TR (1958) Some observations on the natural history of *Acanthaspis* *petax* Stal (Hemiptera: Reduviidae) living in termite mounds in Uganda. Proceedings of the Royal Entomological Society of London (A) 33: 167-175.

117. Costa Lima Ad (1940) Sobre as especies de *Spiniger* (Hemiptera : Reduviidae). Memorias Do Instituto Oswaldo Cruz 35: pp. 1-123.

118. Fritzsche I (2008) Predatory bugs of the genus *Platymeris* Laporte, 1833 (Heteroptera: Reduviidae). Arthropoda 16: 22-27.

119. Edwards JS (1962) Observations on the development and predatory habit of two Reduviid Heteroptera, *Rhinocoris* *carmelita* Stal and *Platymeris* *rhadamanthus* Gerst. Proceedings of the Royal Entomological Society of London (A) 37: 89-98.

120. Immel R (1954) Biologische Beobachtungen an der Staubwanze (*Reduvius* *personatus* L.) (Vorlaufige Mitteilung). Zoologischer Anzeiger Leipzig 152: 96-98.

121. Wood SF (1954) Experimental destruction of the conenose bug *Triatoma* by the assassin bugs, *Reduvius* *personatus* and *R. senilus* (Hemiptera. Reduviidae). Bull Southern California Acad Sci 53: 174-176.

122. Vennison SJ, Ambrose DP (1990) Biology of an Assassin Bug *Velitra* *sinensis* Walker Insecta Heteroptera Reduviidae from South India. Indian Journal of Entomology 52: 310-319.

123. Gnaspini P (1996) Population ecology of *Goniosoma* *spelaeum*, a cavernicolous harvestman from south-eastern Brazil (Arachnida: Opiliones: Gonyleptidae). Journal of Zoology 239: 417-435.

124. Ferreira RL, Martins RP (1999) Trophic structure and natural history of bat guano invertebrate communities, with special reference to Brazilian caves. Tropical Zoology 12: 231-252.

125. Weirauch C, Forero D (2007) *Kiskeya* *palassaina*, new genus and new species of Saicinae (Heteroptera : Reduviidae) from the Dominican Republic. Zootaxa: 57-68.

126. Vennison SJ, Ambrose DP (1987) Predatory behavior of two assassin bugs *Edocla* *slateri* and *Oncocephalus* *annulipes*. Environment and Ecology (Kalyani) 5: 234-238.

127. Villiers A (1948) Hemipteres Reduviides de l'Afrique Noire. Faune de l'Empire Francais 9: 1-488.

128. Ishikawa T, Okajima S (2004) A new species of the saicine assassin bug genus *Carayonia* *villiers* (Heteroptera : reduviidae) from indochina. Proceedings of the Entomological Society of Washington 106: 319-323.
